# Clinical phenotypes of comorbidities in end-stage knee osteoarthritis: a cluster analysis

**DOI:** 10.1186/s12891-024-07394-1

**Published:** 2024-04-17

**Authors:** Jun Ma, Kai Zhang, Xilong Ma, Hao Wang, Chao Ma, Yahui Zhang, Ruiyu Liu

**Affiliations:** 1 Department of Orthopedics, The Second Affiliated Hospital, Xi’an Jiaotong University, Xi’an, Shaanxi China; 2 Ningxia Medical University Third Clinical Medical School, Yinchuan City, Ningxia China; 3 Department of Orthopaedics, People’s Hospital of Ningxia Hui Autonomous Region, Yinchuan City, Ningxia China

**Keywords:** Osteoarthritis, Actor analysis, Cluster analysis, Comorbidity, Phenotypes

## Abstract

**Objectives:**

Comorbidities, as components of these heterogeneous features, often coexist with knee osteoarthritis, and are particularly prevalent in end-stage knee osteoarthritis. Here, we attempted to identify the different clinical phenotypes of comorbidities in patients with end-stage knee osteoarthritis by cluster analysis.

**Methods:**

A total of 421 inpatients diagnosed with end-stage knee osteoarthritis who underwent inpatient surgery were included in this cross-sectional study. 23 demographic, comorbidity, inflammatory immune and evaluation scale variables were collected. Systematic clustering after factor analysis and separate two-step cluster analysis were performed for individual comorbidity variables and all variables, respectively, to objectively identify the different clinical phenotypes of the study patients.

**Results:**

Four clusters were finally identified. Cluster 1 had the largest proportion of obese patients (93.8%) and hypertension was common (71.2%). Almost all patients in cluster 2 were depressed (95.8%) and anxiety disorders (94.7%). Cluster 3 combined patients with isolated end-stage knee osteoarthritis and a few comorbidities. Cluster 4 had the highest proportion of patients with rheumatoid arthritis (58.8%).

**Conclusions:**

Patients with end-stage knee osteoarthritis may be classified into four different clinical phenotypes: "isolated end-stage knee osteoarthritis"; "obesity + hypertension"; "depression + anxiety"; and "rheumatoid arthritis", which may help guide individualized patient care and treatment strategies.

## Introduction

Knee osteoarthritis (KOA) is a chronic inflammatory and degenerative musculoskeletal disease whose incidence, prevalence, and years lived with disability are increasing every year, causing significant personal, economic, and social costs [1, 2]. Despite the great therapeutic success of KOA, approximately 10% of patients are still dissatisfied with their outcome [3]. For this reason, it is increasingly recognized that KOA is a complex and highly heterogeneous disease, consisting of different phenotypes or subtypes with different characteristics at different stages of disease progression [4, 5]. Comorbidities, as components of these heterogeneous features, often coexist with KOA and are particularly prevalent in end-stage KOA. The impact of comorbidities on end-stage KOA is extensive and includes increased health care use and costs, impaired physical and mental health, increased mortality, and reduced quality of life [6–8]. Therefore, it is important to identify the complex relationship between end-stage KOA and comorbidities.

However, awareness and interest in end-stage KOA comorbidity has been poorly demonstrated, with only a few studies reporting on OA comorbidity, such as a recent paper based on a large UK primary care database by Swain et al [9] who examined the temporal relationship of OA comorbidity and found that of 49 comorbidities, 38 of them were associated with OA before and after diagnosis of OA, with only dementia and systemic lupus erythematosus were associated after the diagnosis of OA, and these studies on OA comorbidity are still in the preliminary stage [10]. To date, no attempt has been made to describe how comorbidities are combined in end-stage KOA. Cluster analysis is an ideal method to identify new patterns in data, which divides data objects into different subgroups based mainly on similarity or dissimilarity, and these subgroups can help to understand different pathological mechanisms of the disease [11–13]. We propose to use a two-step clustering algorithm to assess comorbidity interrelationships in end-stage KOA and identify different clusters of patients with end-stage KOA comorbidity with the aim of informing the management and personalized treatment of end-stage KOA patients with comorbidity.

## Methods

### Data sources

A cross-sectional single-center study design was used to select inpatients diagnosed with end-stage KOA at our institution between December 1, 2021 and November 30, 2022, and participants who met the inclusion criteria were approached to determine their level of interest in participating in the survey. Questionnaires and access to the inpatient electronic medical record and laboratory tests were administered to patients with a high level of interest and who had obtained written informed consent. Demographics (age, sex, height, weight and abdominal circumference), laboratory tests (neutrophil count, lymphocyte count, erythrocyte sedimentation rate (ESR), uric acid, fasting glucose, blood lipids, creatinine, liver enzymes and ultrasensitive C-reactive protein (hs-CRP)), ECG, abdominal ultrasound, questionnaire scales (EQ-VAS [14], HADS [15] and VRS scales [16]), KOA history (pain duration, pain frequency and side) and comorbidities (obesity, hypertension, type 2 diabetes, coronary artery disease, hyperlipidemia, stroke, sleep disorders, depression, anxiety, hyperuricemia, chronic kidney disease, liver disease and rheumatoid arthritis).

### Inclusion & exclusion criteria

Inclusion criteria: (1) patients with a confirmed clinical and imaging diagnosis of end-stage KOA (Kellgren-Lawrence, K-L=class IV [17] ); (2) well-documented clinical profile; (3) age ≥ 45 years; (4) stable condition of patients (stable vital signs, no potential risk of deterioration and life-threatening conditions).

Exclusion criteria: (1) history of previous joint trauma or knee surgery, or presence of joint disease such as septic arthritis or simple rheumatoid arthritis; (2) patients with serious complications during hospitalization (coma, acute coronary syndrome, MODS, diabetic ketoacidosis, systemic infection, etc.); (3) patients who were unwilling to participate in communication or had mental disorders and other inability to cooperate; (4) missing clinical data.

### Variable definition and evaluation

Neutrophil count to lymphocyte ratio (NLR) is a novel indicator of inflammation and immune homeostasis that has been gaining recognition in recent years [18]. Patients' pain symptoms and their own health status on the day of hospitalization were assessed using a verbal measurement scale (VRS) and a health self-assessment (EQ-VAS), the former of which is a 5-point scale consisting of five pain levels: no pain, mild pain, moderate pain, severe pain, and extreme pain, from which patients are asked to choose the level that best describes their pain intensity; the latter is part of the EQ-5D-5L scale [19], which consists of a 20 cm vertical line with a score of 0 at the bottom for "worst imaginable health" and 100 at the top for "best imaginable health". The Hospital Anxiety and Depression Scale (HADS) is the most commonly used tool to screen for depression and anxiety disorders and consists of two subscales, anxiety (HADS-A) and depression (HADS-D), with a total of 14 items scoring 0–21, of which ≤ 7 is negative, 8–10 is suspicious, and ≧11 is positive. Pain duration was defined as the time between the onset of intermittent or persistent knee pain and hospitalization for surgery. End-stage knee osteoarthritis combined with rheumatoid arthritis and rheumatoid arthritis alone are defined based on knee radiographs. If the patient was previously diagnosed with rheumatoid arthritis and the knee radiographs showed massive bone formation, significant joint space narrowing at weight-bearing areas and severe subchondral sclerosis, we defined the patient with this radiographic presentation as having end-stage knee osteoarthritis combined with rheumatoid arthritis. If the radiographs of the knee joint showed diffuse and uniform narrowing of the joint space (weight-bearing and non-weight-bearing areas) and erosive destruction of the subchondral bone with severe periarticular osteoporosis, without significant bone redundancy and subchondral osteosclerosis, we defined patients with this radiological feature as having simple rheumatoid arthritis, and these patients were excluded from our study population. Variables were intentionally limited to demographic, comorbidity, inflammatory immune and evaluation scale variables including obesity, hypertension, type 2 diabetes, coronary artery disease, hyperlipidemia, stroke, sleep disorders, depression, anxiety, hyperuricemia, chronic kidney disease, liver disease, rheumatoid arthritis, ESR, hs-CRP, NLR, VRS, EQ-VAS, BMI, abdominal circumference, age gender, and pain duration. Among the comorbidity variables were selected common comorbidities associated with KOA as reported in previous literature [20, 21]. The names and specific definitions of the comorbidity variables are shown in Table 1.

**Table 1:** Definition of comorbidity variables

Comorbidities	Definition
Obesity	BMI ≥ 28 kg/m^2^
Hypertension	Systolic blood pressure ≥ 140 mmHg and or diastolic blood pressure ≥ 90 mmHg or history of hypertension
Type 2 diabetes	Fasting blood glucose ≥ 7.0 mmol/L or HbA1c ≥ 6.5 or history of type 2 diabetes
Stroke	Pre-existing diagnosis of stroke
Hyperlipidemia	TC ≥ 6.22 mmol/L or TG ≥ 2.26 mmol/L
Sleep disorder	Insomnia ≥ 3 times per week for more than 1 month
Hyperuricemia	Fasting blood uric acid ≥ 420 umol/L or previous history of hyperuricemia
Coronary artery disease	Prior history of angina pectoris and/or myocardial infarction or ECG-confirmed coronary artery disease
Depression	D(HADS) ≧ 11 or previous diagnosis of depression
Anxiety	A(HADS) ≧ 11 or previous diagnosis of anxiety disorder
Chronic kidney disease	Blood creatinine ≥ 133umol/L or previous history of chronic kidney disease
Liver disease	Hepatic steatosis or cirrhosis or abnormal liver enzyme levels (ALT ≥ 60 U/L)
Rheumatoid arthritis	Previously diagnosed rheumatoid arthritis (excluding simple rheumatoid arthritis)

### Statistical analysis

Continuous variables are expressed as mean ± standard deviation. Categorical variables are expressed as frequencies and percentages (%). Continuous variables were compared by Student t-test or ANOVA, and categorical variables were compared by χ2 test or Fisher's exact test. Two-tailed *p* values less than 0.05 were considered statistically significant. All data were statistically analyzed using IBM SPSS.26.0.

We conducted two clustering analyses using systematic clustering and a separate two-step Cluster Algorithm. In the first analysis, we used factor analysis to combine multiple comorbidity variables into a few factors that reflect commonality. We employed the principal component method in factor analysis to find the most suitable factor loading matrix, which was then rotated using the maximum variance method. These factor matrices were systematically clustered using Ward's method, and the results were represented in a tree diagram showing the variables in each grouping and the distance between the groups. In the second analysis, we normalized continuous variables, removed anomalous noise points, and used log similarity values as distance measures between variables. The BIRCH algorithm was utilized to construct and modify the Clustering Feature Tree (CFT) for pre-clustering, and the pre-clustered results were further clustered using the log likelihood function. The final output of our data analysis is presented as a model (22, 23).

## Results

### Population

This study included 493 patients with diagnosed end-stage knee osteoarthritis, all of whom were hospitalized between November 1, 2021, and November 30, 2022, for treatment with artificial total knee arthroplasty; 72 patients were excluded, including (1) those with a history of prior knee surgery (including contralateral artificial total knee arthroplasty) (*n* = 29); (2) those with incomplete data (missing hs-CRP and ESR) (*n* = 2); (3) those who underwent contralateral knee arthroplasty again during the questionnaire period (*n* = 21); (4) refusal to accept the questionnaire (*n* = 16); (5) traumatic knee osteoarthritis (*n* = 2); (6) combined severe congenital hip dysplasia (*n* = 1), 421 patients (87 men and 334 women) with sufficiently complete clinical data and met all inclusion criteria were screened for final study analysis. The majority of patients were female (79%), aged from 48 to 86 years, with a mean age of 67 ± 6 years, and waist circumference was greater in females than in males (90.6 ± 7.8 cm vs. 87.0 ± 10.4 cm, *p* < 0.05). In addition, the majority of patients were diagnosed with bilateral knee osteoarthritis (65%), almost twice the proportion of unilateral knee osteoarthritis (35%). Nearly four-fifths (87%) of patients presented with intermittent knee pain, significantly higher than persistent knee pain (13%). The mean duration of knee pain was 11.3 ± 7.3 years, with no statistically significant difference between women and men (*p* = 0.211) (Table 2).

**Table 2:** Characteristics of patients with end-stage KOA (*n* = 421)

Characteristic	Total *n* = 421	Men *n* = 87	Women *n* = 334	*P* Value
Age (years)	67 ± 6	68 ± 7	67 ± 6	0.127
BMI (kg/m^2^)	25.3 ± 3.4	24.7 ± 3.5	25.5 ± 3.3	0.497
Waist circumference (cm)	89.5 ± 9.4	87.0 ± 10.4	90.6 ± 7.8	**0.010**
Side				
Single	147 (35)	32 (37)	115 (34)	0.682
Double	274 (65)	55 (63)	219 (66)	
Nature of pain				
Intermittent	365 (87)	74 (85)	291 (87)	**0.000**
Constant	56 (13)	13(15)	43 (13)	
Symptom duration (years)	11.3 ± 7.3	9.9 ± 7.0	11.7 ± 7.3	0.211
Comorbidity				
Obesity	113 (27)	19 (22)	94 (28)	0.237
Hypertension	220 (52)	36 (41)	184 (55)	**0.023**
Diabetes	97 (23)	14 (16)	83 (25)	0.084
Coronary heart disease	70 (17)	11 (13)	59 (18)	0.263
Hyperuricemia	40 (10)	4 (5)	36 (11)	0.100*
Stroke	43(10)	9(10)	34(10)	0.964
Rheumatoid arthritis	25(6)	3(3)	22(7)	0.443*
Chronic renal disease	4(1)	0(0)	4(1)	0.585*
Liver disease	123(29)	20(23)	103(31)	0.152
Sleep disorders	40(10)	30(34)	10 (3)	0.477
Depression	143 (34)	19 (22)	124 (37)	**0.007**
Anxiety	109 (26)	15 (17)	94 (28)	**0.039**
Hyperlipidemia	59(14)	6(2)	53(61)	**0.032**

Data are presented as mean ± standard deviation for continuous variables, and categorical variables are expressed as frequencies and percentages (%); Student t test was used for continuous variables and χ2 test was used for dichotomous variables. Two-tailed *p* values less than 0.05 were considered statistically significant

^*^Fisher exact test

### Comorbidities in the study population

Of the 13 common comorbidities in 421 end-stage KOA, 64 (15%) patients had no comorbidity, 78 (19%) patients had 1 comorbidity, 156 (37%) patients had 2–3 comorbidities, and 123 (29%) patients had more than 3 comorbidities. The most common comorbidities were: hypertension (52%), depression (34%), liver disease (29%), obesity (27%) and anxiety disorders (26%). Notably, the prevalence of hypertension (*p* = 0.023), depression (*p* = 0.007), anxiety (*p* = 0.039) and hyperlipidemia (*p* = 0.032) was higher in women than in male patients (Table 2).

### Comorbidities age distribution characteristics

Age is a strong correlate common to most chronic diseases, and by grouping different age groups, we investigated whether the prevalence of comorbidities changed with age (Table 3). The results found that BMI and abdominal circumference were not associated with increasing age (*p* > 0.05). The prevalence of hypertension and coronary heart disease increased with age (*p* < 0.05), and the prevalence of stroke also showed age-specific differences, with the highest prevalence over 70 years of age (*p* = 0.006). In contrast, the prevalence of type 2 diabetes, obesity, chronic kidney disease, hyperlipidemia, hyperuricemia, liver disease, depression, anxiety, sleep disorders, and rheumatoid arthritis were not associated with increasing age.

**Table 3:** Comorbidities age distribution with end-stage knee osteoarthritis

Characteristic	Age Segments (years)			*P* Value
	< 60	60–70	> 70	
N	51	234	136	*-*
Demographics				
BMI (kg/m^2^)	24.9 ± 2.9	25.4 ± 3.3	25.3 ± 3.7	0.388
Female gender	41 (80)	192 (82)	101 (74)	-
Waist circumference (cm)	87.7 ± 8.7	90.0 ± 9.0	90.0 ± 10.3	0.115
Comorbidity				
Obesity	13 (26)	63 (27)	37 (27)	0.972
Hypertension	24 (47)	112 (48)	84 (62)	**0.026**
Diabetes	11 (22)	53 (23)	33 (24)	0.906
Coronary heart disease	2 (4)	39 (17)	29 (21)	0.010*
Hyperuricemia	4 (8)	22 (9)	14 (10)	0.916*
Stroke	5(10)	15(6)	23(17)	**0.006**
Rheumatoid arthritis	5(10)	16(7)	4(3)	0.130*
Chronic renal disease	0(0)	1(0)	3(2)	0.175*
Liver disease	14(28)	69(30)	40(29)	0.957
Sleep disorders	7(14)	18(8)	15 (11)	0.314
Depression	19 (37)	80 (34)	44 (32)	0.815
Anxiety	15 (30)	58 (25)	36 (27)	0.778
Hyperlipidemia	6(12)	34(15)	19(14)	0.875

Data are presented as mean ± standard deviation for continuous variables, and categorical variables are expressed as frequencies and percentages (%); Student t test was used for continuous variables and χ2 test was used for dichotomous variables. Two-tailed *p* values less than 0.05 were considered statistically significant

^*^Fisher exact test

### Clustering of comorbidity variables

**Fig. 1:**
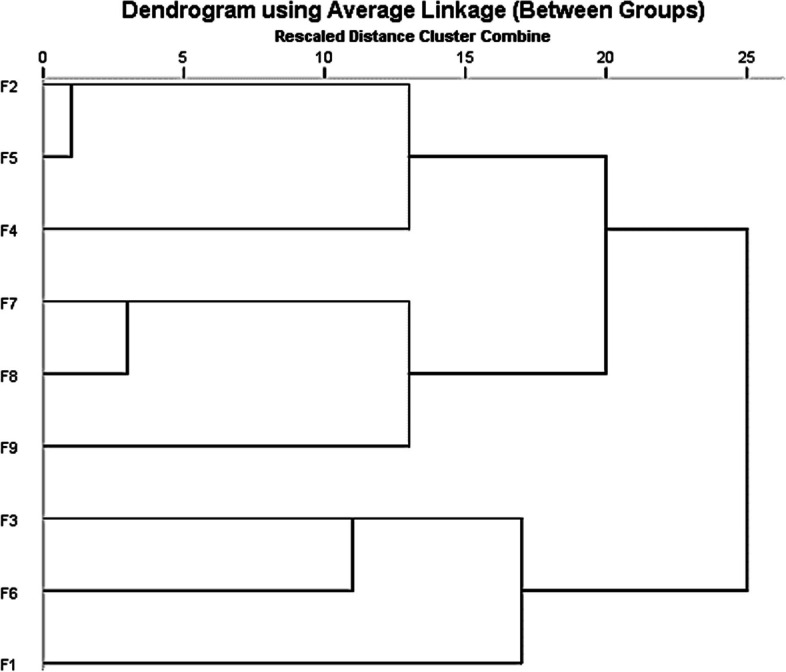
Dendrogram of the results of cluster analysis of comorbidity variables(9 common factors). Systematic cluster analysis was used to classify the 9 common factors. Variables with similar response patterns were grouped together, and variables with different response patterns were separated. Each horizontal line represents an individual variable, and the length of the horizontal line represents the degree of similarity between the variables

To better assess the correlation between variables, two independent cluster analyses were performed. The initial clustering was limited to comorbidity variables, and considering the redundancy between comorbidities, we performed a factor analysis. The first 4 factors were extracted based on eigenvalues > 1, and their cumulative variance contribution to explain the 13 comorbidity variables accounted for only 48% (Table 4). Therefore, we finally selected the top 9 factors with a total cumulative variance contribution of 83% (eigenvalue > 0.8). The factor loading matrix was rotated using the great variance method, and the results in Table 5 were obtained: F1 was associated with psychosomatic disorders (depression and anxiety); F2 was associated with metabolic syndrome (hyperlipidemia and type 2 diabetes); F3 was associated with abnormal fat metabolism (liver disease and obesity); F4 was associated with hyperuricemia; F5 was associated with cardiovascular disease (hypertension and stroke); F6 was associated with coronary heart disease; F7 was associated with rheumatoid arthritis; F8 was associated with chronic kidney disease; and F9 was associated with sleep disorders. The results of the correlation matrix of comorbidity variables were analyzed by systematic clustering, and four clusters were obtained (Fig. 1), including C1: strong association between metabolic syndrome and cardiovascular disease; C2: association between rheumatic diseases and chronic kidney disease; C3: association between abnormal fat metabolism and coronary heart disease; and C4: psychosomatic diseases appeared to be independent.

**Table 4:** Factors explaining the total variance of the original influences

Total Variance Explained						
Initial Eigenvalues				Extraction Sums of Squared Loadings		
Component	Total	% of Variance	Cumulative %	Total	% of Variance	Cumulative %
1	2.131	16.390	16.390	2.131	16.390	16.390
2	1.833	14.103	30.493	1.833	14.103	30.493
3	1.253	9.635	40.128	1.253	9.635	40.128
4	1.081	8.315	48.443	1.081	8.315	48.443
5	0.979	7.533	55.976			
6	0.919	7.073	63.048			
7	0.913	7.026	70.074			
8	0.877	6.744	76.818			
9	0.809	6.222	83.040			
10	0.715	5.499	88.539			
11	0.665	5.116	93.655			
12	0.522	4.017	97.672			
13	0.303	2.328	100.000			

Extraction method: principal component analysis

**Table 5:** Factor loading matrix after rotation

Rotated component matrix^a^	Component								
	1	2	3	4	5	6	7	8	9
Anxiety	0.906	0.006	0.043	-0.022	0.042	0.016	0.067	0.035	0.114
Depression	0.896	-0.002	-0.016	0.013	0.086	0.077	0.094	0.020	-0.020
Hyperlipidemia	0.004	0.813	-0.067	0.143	-0.060	0.054	-0.158	-0.184	-0.004
Diabetes	-0.008	0.797	.220	-0.082	0.037	0.038	0.190	0.186	-0.026
Liver disease	-0.045	0.051	.906	-0.097	0.026	0.081	0.068	0.018	-0.036
Obesity	0.212	.190	.565	0.379	-0.058	0.034	-0.319	-0.214	-0.008
Hyperuricemia	-0.033	.050	-.027	0.928	0.054	0.045	0.066	0.118	-0.001
Stroke	0.105	-.059	-.026	0.009	0.931	-0.038	0.013	-0.029	0.089
Hypertension	0.048	.377	.129	0.187	0.432	0.351	-0.141	0.157	-0.072
Coronary Heart Disease	0.079	0.067	0.075	0.030	-0.009	0.960	-0.029	-0.021	0.054
Rheumatoid arthritis	0.168	0.006	-0.006	0.057	-0.025	-0.044	0.921	-0.067	0.027
Chronic Kidney Disease	0.053	0.006	-0.046	0.103	0.002	-0.003	-0.058	0.942	0.034
Sleep disorders	0.083	-0.030	-0.038	-0.003	0.070	0.044	0.027	0.034	0.985

Extraction Method: principal component analysis

Rotation Method:Varimax with Kaiser Normalization

^a^Rotation converged in 6 iterations

### Clustering model and size

**Fig. 2:**
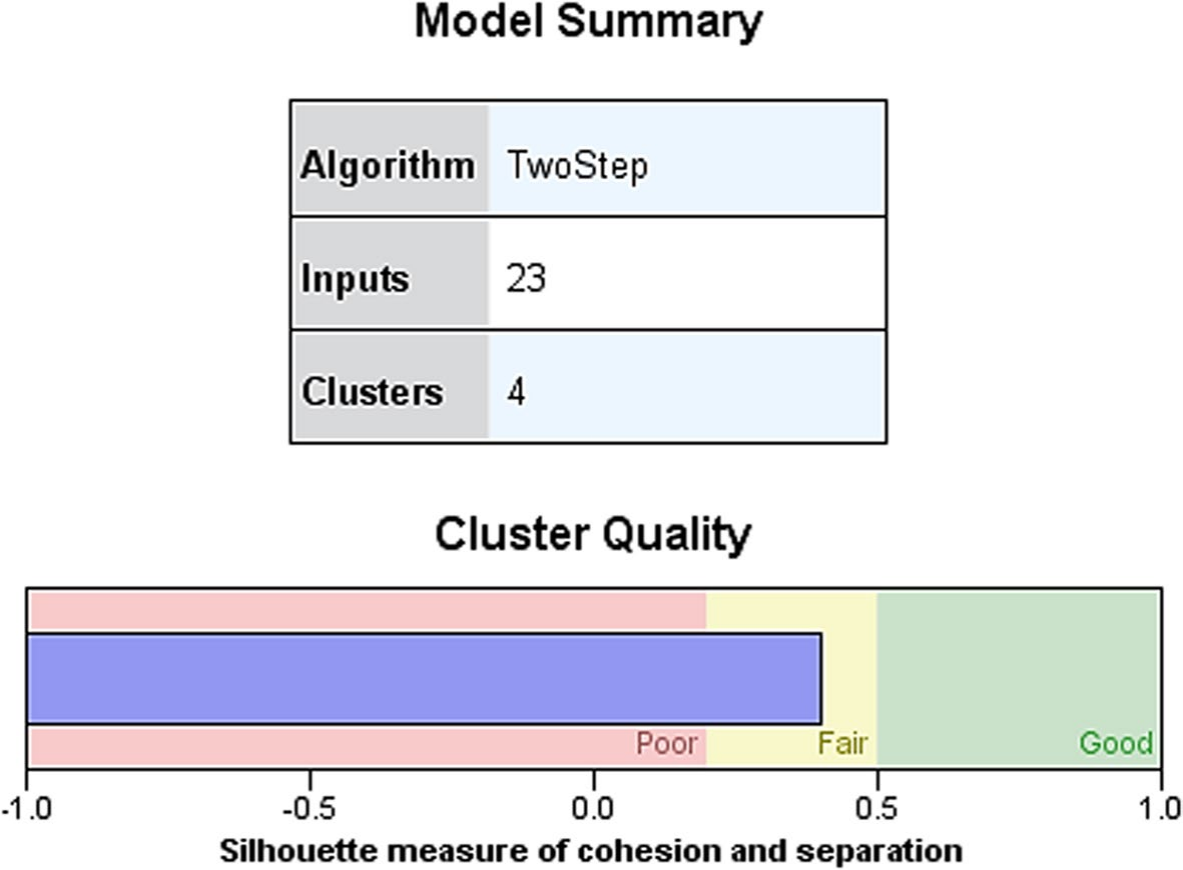
Clustering quality

**Fig. 3:**
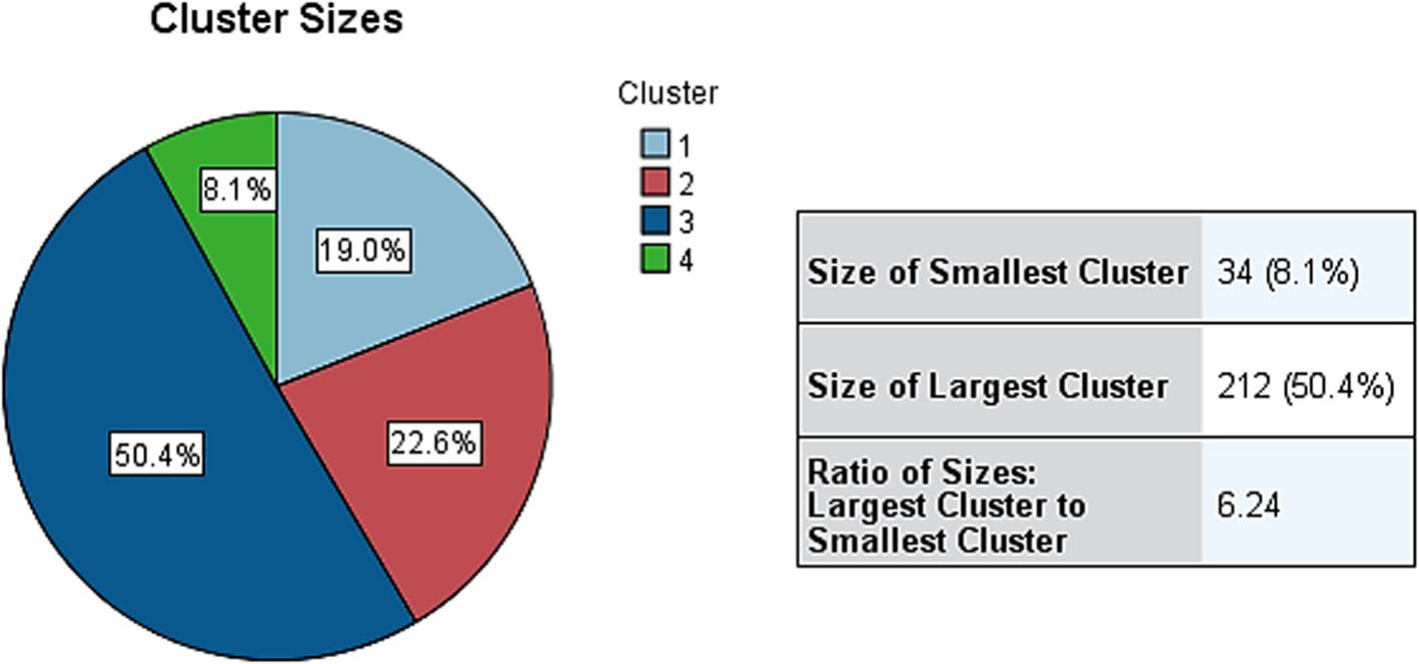
Clustering size

We then classified 421 subjects with end-stage knee osteoarthritis comorbidities using a two-step cluster analysis. Variables included demographic, comorbidity, inflammatory immune and evaluation scale variables, with a total of 23 variables (9 continuous and 14 categorical) entered, including obesity, hypertension, type 2 diabetes, coronary artery disease, hyperlipidemia, stroke, sleep disorders, depression, anxiety, hyperuricemia, chronic kidney disease, liver disease, rheumatoid arthritis, ESR, hs-CRP, NLR, VRS, EQ-VAS, BMI, abdominal circumference, age, gender, and pain duration. Four clusters were eventually generated with good quality of clustering (Fig. 2). Cluster 3 was the largest cluster, including 212 patients or 50.4%; cluster 4 was the smallest cluster, including 34 patients or 8.1%; cluster 1 was the third largest cluster, including 80 patients or 19.0%; and cluster 2 was the second largest cluster, including 95 patients or 22.6% (Fig. 3).

### Predictive variables importance

**Fig. 4:**
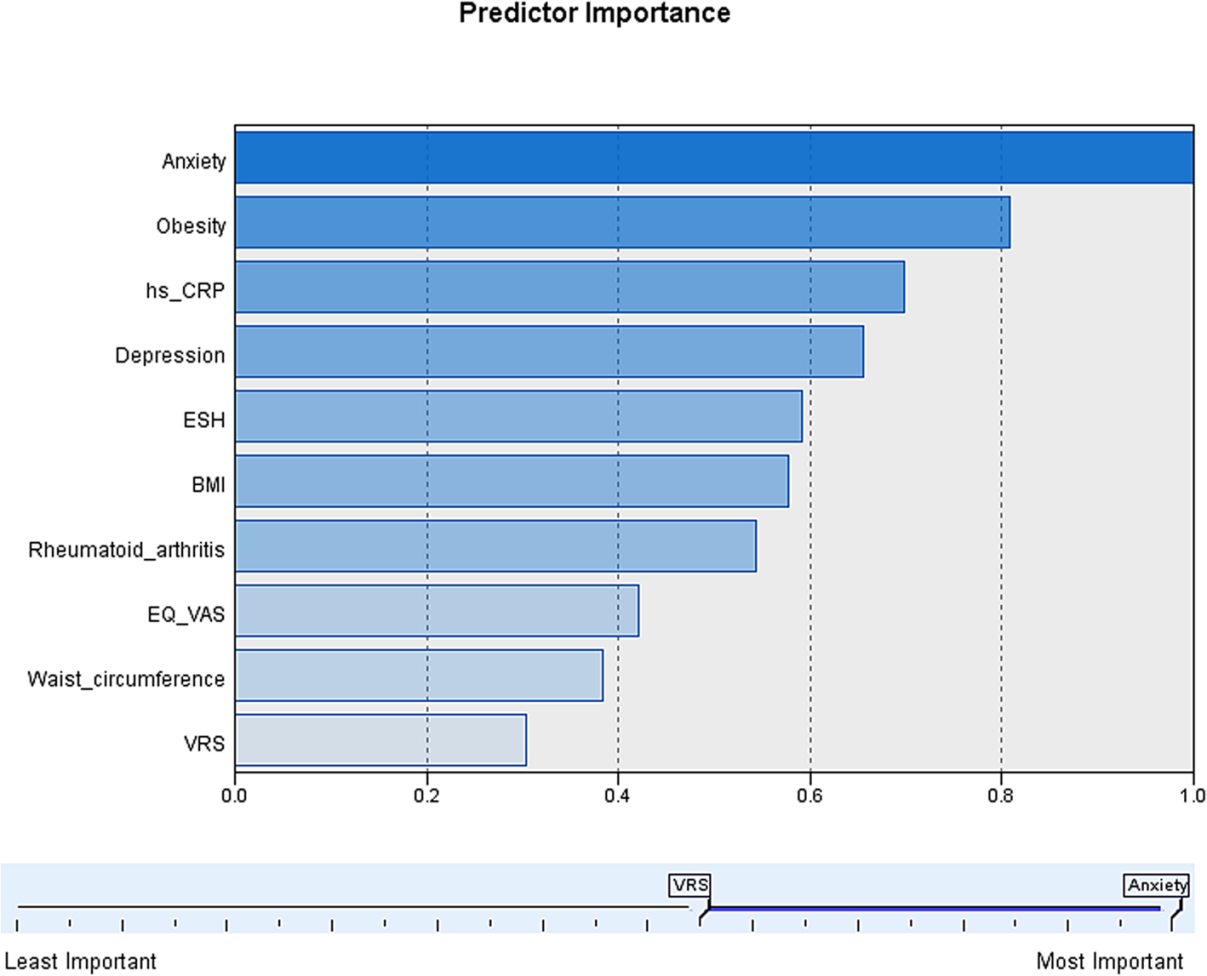
Predictive variables importance ranking

Among the importance of predictor variables for end-stage KOA comorbidity, anxiety was the most important with a value of 1.0, followed by obesity (0.81), ultrasensitive C-reactive protein (0.70), depression (0.66), blood sedimentation (0.59), BMI (0.58) and rheumatoid arthritis (0.54) (Fig. 4).

### Clustering overall distribution

Cluster 1 (*n* = 80, 19%) had the largest proportion of obese patients (93.8%). Hypertension was also common (71.2%). BMI, abdominal circumference, hyperlipidemia, coronary heart disease, liver disease, hyperuricemia, and type 2 diabetes mellitus had the highest proportions in all clusters. As with C3, EQ-VAS and VRS scores were better. In addition, this group had the fewest patients with sleep disorders and none had chronic kidney disease.

Cluster 2 (*n* = 95, 22.6%) differed from C3 mainly in that almost all patients were depression (95.8%) and anxiety (94.7%). Hypertension were also common (61.1%). As in Cluster 4, EQ-VAS and VRS scores were poor and sleep disorders were the most prominent problem. The highest percentage of female patients (84.2%) and the highest prevalence of stroke (22.1%) were also observed. Notably, the shortest mean duration of pain (10.13 years) was observed in this group.

Cluster 3 (*n* = 212, 50.4%) combined patients with isolated end-stage KOA and few comorbidities. The highest percentage of men (24.1%) had the lowest inflammation-related indicators, and none were obese or had anxiety.

**Fig. 5:**
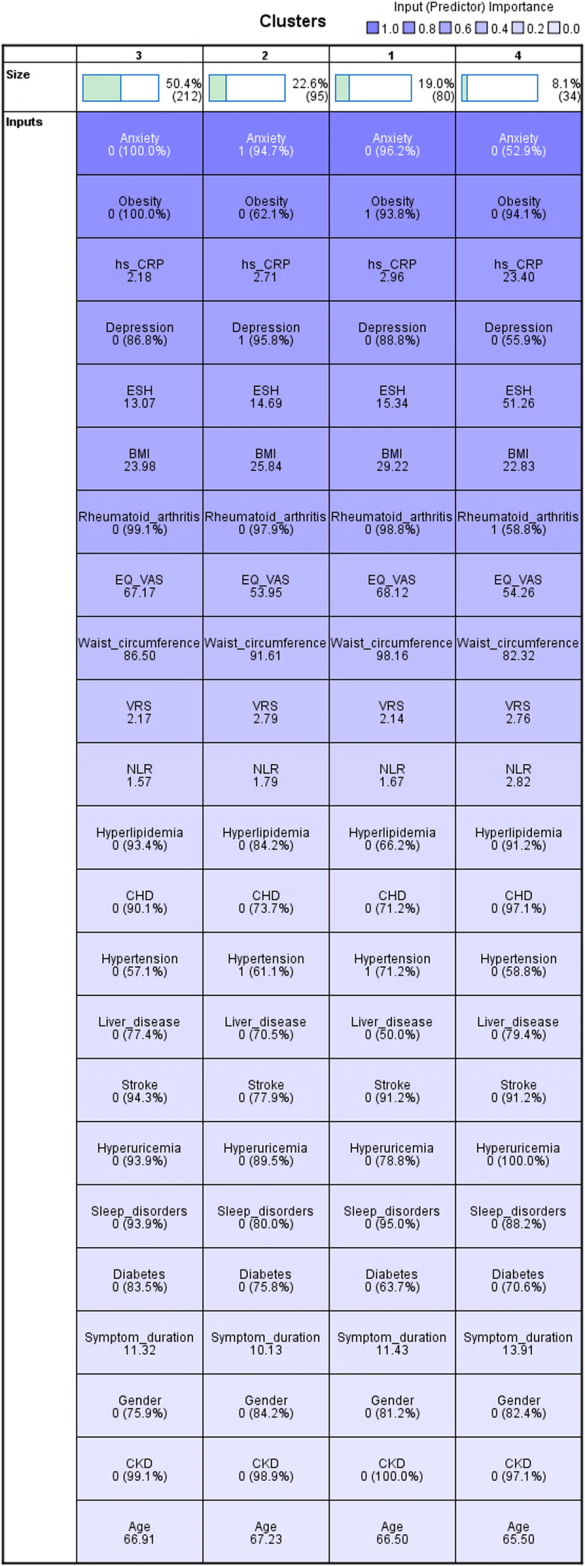
Results of cluster analysis of all study variables. occurrence of comorbidities (yes = 1, no = 0, expressed as percentage (%))

Cluster 4 (*n* = 34, 8.1%) had the highest proportion of patients with rheumatoid arthritis (58.8%). the smallest BMI (22.83) and abdominal circumference (82.32), worse EQ-VAS (51.26) and VRS (2.76) scores, the longest mean duration of pain (13.91 years), and the highest blood sedimentation (49.16), ultrasensitive C-reactive protein (23.40) and neutrophil count/lymphocyte count ratio (2.82) were the highest. Notably, sleep disorder problems (11.8%) were more prominent in this group, and the prevalence of chronic kidney disease was the highest of all groups (2.9%). Almost no one had coronary heart disease (2.9%) and no one had hyperuricemia disease (Fig. 5).

## Discussion

Overall, individuals with end-stage KOA often had comorbidities with other chronic diseases, as the prevalence of at least 1 comorbidity in our sample was 85%, significantly higher than the 15% prevalence in individuals without comorbidities. Our findings are consistent with previous cohort studies, but with a higher proportion of comorbidities of 1 or more comorbidities. For example, a retrospective study conducted in Canada in 2019 showed that 54.6% of patients with OA had at least one of eight comorbidities; another meta-analysis based in 16 countries also found that 67% of patients with OA had at least one comorbidity [20, 21]. This may be related to the inclusion of our study population in the end-stage of knee osteoarthritis, as age is a strong correlate of the prevalence of most chronic diseases, and the onset of chronic disease is associated with its accumulation of temporal exposure to associated risk factors and changes in the declining function of the body. Furthermore, with regard to the most common comorbidities, our results were similar to other previously published cohort studies in that psychological disorders, cardiovascular disorders and metabolic syndrome comorbidities were the most common, with the exception of liver disease [9, 20]. We believe that the reason for the high proportion of liver disease is related to the definition describing this comorbidity, with the highest proportion of liver diseases in our cohort being non-alcoholic fatty liver disease, which has a high clinical prevalence and a high number of co-pathogenic factors with end-stage KOA [22]. Notably, we tapped depression and obesity as the most important predictor variables of end-stage KOA comorbidity. Numerous studies have consistently shown a notable association between depression and obesity, which leads to a higher incidence of chronic diseases. This association follows a positive dose–response relationship, indicating that depression and obesity may potentially act as co-causal risk factors for end-stage KOA and comorbidity aggregation. Furthermore, depression is linked to chronic, low-grade inflammation, cell-mediated immune activation, and the activation of compensatory anti-inflammatory reflex systems [23, 24].

It is well known that age is a strong correlate common to most chronic diseases, and we suggest that the prevalence of end-stage KOA comorbidities is associated with increasing age. By stratifying age, we found that the prevalence of hypertension and coronary heart disease increased with age. Stroke prevalence also showed age-specific differences, with the highest prevalence over 70 years of age. This result also supports the existence of a temporal longitudinal relationship between end-stage KOA and cardiovascular disease aggregation, and possible explanations for this, in addition to differences in chronic low-grade inflammation and low estrogen levels, end-stage KOA-related walking impairment and long-term NSAID use may be major factors [25, 26].

Considering the characteristics of the data variables in this study, we finally chose a two-step cluster analysis. A total of 4 comorbidity clusters were identified, each containing a different number of clusters, and each cluster, except for cluster 3, combined 1–2 diseases with relatively high proportions.

Cluster 3, the largest cluster, was characterized by isolated end-stage knee osteoarthritis. Interestingly, this group showed the lowest indicators of inflammation compared to the other clusters with combined comorbidities, suggesting that inflammation may act as a risk factor for the aggregation of comorbidities in end-stage KOA. Furthermore, the age of the patients in this cluster was similar to the other groups, indicating that different clusters are associated with specific phenotypes.

Patients in cluster 2 experienced poorer health status and more pain, along with significant sleep disorders. It is widely known that depression and anxiety often coexist with end-stage KOA disease, but the underlying pathophysiology remains unclear. Possible mechanisms include psychosocial effects, immune-inflammatory activation, epigenetic modifications, and alterations in structural brain function [27, 28]. Additionally, this group had the highest prevalence of stroke (22.1%), which aligns with previous reports. For instance, anxiety disorders can impact the resistance of vascular endothelial and smooth muscle function, leading to increased prevalence of hypertension and stroke. Depression and anxiety are also commonly observed after stroke, with prevalence rates ranging from approximately 29% to 55%. It is now believed that brain-derived neurotrophic factor (BDNF) may play a key role in these associations [29, 30]. Notably, this group had the shortest mean pain duration (10.16 years), which we believe is linked to more severe pain experiences, poorer health, and heightened concerns about the disease, as these factors are more likely to prompt patients to seek treatment earlier.

Cluster 1, reflecting end-stage KOA combined with metabolic syndrome, had a high prevalence of obesity, hypertensive disease, diabetes mellitus, liver disease, hyperuricemia, and hyperlipidemia. These findings support the concept of metabolic phenotypes as clinical manifestations of OA [31]. Interestingly, our results indicate that the metabolic syndrome did not have a significant impact on pain, health status, and sleep disturbances in patients with end-stage.

KOA.Cluster 4 consisted mainly of patients with rheumatoid arthritis (58.8%), with the lowest BMI (22.83) and abdominal circumference (82.32 cm), which explains the low prevalence of comorbid coronary artery disease in this group (2.9%). Similar to cluster 2, patients in this cluster had the poorest health status and experienced the most pain, along with a notable problem of sleep disorders (11.8%). These findings align with previous studies that have identified high levels of inflammatory state and immune system dysregulation as the main underlying mechanisms (ESR 286 (49.16), hs-CRP (23.40), and NLR (2.82)) [18, 32]. Additionally, these causative factors are also associated with a higher prevalence of chronic kidney disease (2.9%) [33]. It is worth noting that, unlike cluster 2, this group had a longer mean pain duration (13.91 years), but both clusters reported similar pain levels (VRS: 2.76 vs. 2.79). Therefore, we hypothesize that patients with depressive and anxious psychiatric disorders may be more inclined to undergo surgery earlier, even with less severe pain experiences.

Our study has some limitations. First, the selection of patients was nonrandomized, while the disease study was stage- and site-specific, which allowed limited extrapolation of patient sample results. Second, our definitions of some of the comorbidities were derived from patients' recollections, which may be less accurate or have the potential for underdiagnosis. Finally, we performed only a single clinical phenotypic analysis, which cannot explain the mechanism of action between end-stage KOA comorbidities in detail at the molecular genetic level.

## Conclusion

In conclusion, to the best of our knowledge, this study is the first attempt to perform subgroup analysis of end-stage KOA comorbidities using a clustering approach, and we finally identified four phenotypes, including C1: isolated end-stage knee osteoarthritis; C2: depression + anxiety; C3: obesity + hypertension and C4: rheumatoid arthritis. In addition, this study also provides directions for personalized treatment strategies for patients with end-stage KOA comorbidities, and future combined multi-omics analyses should be performed to elucidate the mechanisms of interactions between end-stage knee osteoarthritis comorbidities.

## Data Availability

The datasets used and/or analyzed during the current study are available from the corresponding author on reasonable request.
